# Graphene oxide decorated with zinc oxide nanoflower, silver and titanium dioxide nanoparticles: fabrication, characterization, DNA interaction, and antibacterial activity†

**DOI:** 10.1039/c8ra09788g

**Published:** 2019-01-28

**Authors:** Nagi El-Shafai, Mohamed E. El-Khouly, Maged El-Kemary, Mohamed Ramadan, Ibrahim Eldesoukey, Mamdouh Masoud

**Affiliations:** Department of Chemistry, Faculty of Science, Alexandria University Egypt; Institute of Nanoscience and Nanotechnology, Kafrelsheikh University Egypt; Department of Chemistry, Faculty of Science, Kafrelsheikh University Egypt; Institute of Basic and Applied Sciences, Egypt-Japan Institute of Science and Technology Alexandria Egypt mohamed.elkhouly@ejust.edu.eg; Department of Bacteriology, Mycology and Immunology, Faculty of Veterinary Medicine, Kafrelsheikh University Egypt

## Abstract

The fabrication, characterization, and antibacterial activity of novel nanocomposites based on graphene oxide (GO) nanosheets decorated with silver, titanium dioxide nanoparticles, and zinc oxide nanoflowers were examined. The fabricated nanocomposites were characterized by various techniques including X-ray diffraction, ultraviolet-visible light absorption and fluorescence spectroscopy, Brunauer–Emmett–Teller theory analysis, Fourier transform infrared, and scanning electron microscopy. The antibacterial activity of the GO–metal oxide nanocomposites against two Gram-positive and two Gram-negative bacteria was examined by using the standard counting plate methodology. The results showed that the fabricated nanocomposites on the surface of GO could inhibit the growth of microbial adhered cells, and consequently prevent the process of biofilm formation in food packaging and medical devices. To confirm the antibacterial activity of the examined GO-nanocomposites, we examined their interactions with bovine serum albumin (BSA) and circulating tumor DNA (ctDNA) by steady-state fluorescence spectroscopy. Upon addition of different amounts of fabricated GO-nanocomposites, the fluorescence intensities of the singlet states of BSA and ctDNA were considerably quenched. The higher quenching was observed in the case of GO–Ag–TiO_2_@ZnO nanocomposite compared with other control composites.

## Abstract 2

The fabrication, characterization, and antibacterial activity of novel nanocomposites based on graphene oxide (GO) nanosheets decorated with silver, titanium dioxide nanoparticles, and zinc oxide nanoflowers were examined.

## Graphical abstract

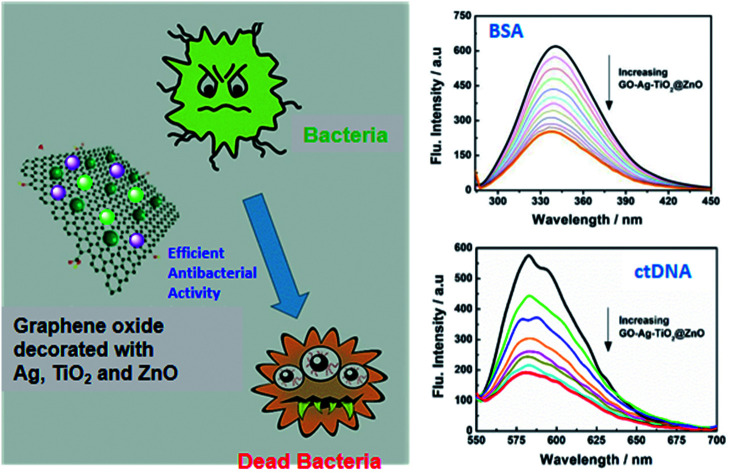

## 1. Introduction

Microorganisms, such as bacteria communities, are found in many different environments (water, soil, skin, and air), and can anchor on various surfaces to produce biofilms that often show high resistance to antimicrobial drugs.^1,2^ In the food industry, the presence of biofilms leads to severe hygiene problems, economic losses, food spoilage, and even serious infectious diseases.^2–5^*Escherichia coli* (*E. coli*) is a bacterial commensal microflora in the intestinal tract of a number of animals, including humans. Although most strains of *E. coli* are harmless, some are able to cause diseases in humans as well as in mammals and birds.^6^*Staphylococcus aureus* (*S. aureus*) is a microorganism that is present as a commensal on the skin, the nose, and mucous membranes of healthy humans and animals. However, it is also an opportunistic pathogen that can cause multiple infectious diseases of varying severity.^7^*Pasteurella multocida* (*P. multocida*), a Gram-negative coccobacillus, is a member of the normal flora of the upper respiratory and gastrointestinal tract of many domestic and wild animals.^8^*Bacillus anthracoides* (*B. anthracoides*) is a group of widely distributed bacteria in nature. Most strains are nonpathogenic; however, some may cause serious infectious diseases in humans and animals.

The resistance of bacteria and fungi to traditional antibiotics is an increasing problem, and the identification and treatment of antibiotic-resistant microorganisms is difficult and costly.^9,10^ Furthermore, complications associated with antibiotic-resistant bacterial infections are a cause of high morbidity and mortality, with antibiotic resistance leading to challenges such as inhibition of drug uptake, enzymatic modification of antibiotics, and alteration of target molecules. Therefore, the development of new antimicrobial drugs based on nanoparticles for the treatment of resistant pathogens may have many advantages, including low toxicity and reduced cost compared with conventional antibiotics.^11–13^ Several studies have examined the antibacterial activity of nanoparticles against both Gram-positive and Gram-negative bacteria, such as iron oxide (Fe_3_O_4_), zinc oxide (ZnO),^14^ copper oxide (CuO),^15^ titanium oxide (TiO_2_),^16^ silver (Ag),^17^ magnesium oxide (MgO),^18^ graphene oxide (GO), reduced graphene (rGO),^19^ nitric oxide (NO) nanoparticles,^20,21^ and carbon nanotubes and graphene, which are chemically modified to graphene oxide^22^ and able to form stable dispersions in water.^23–27^

Over recent decades, silver nanoparticles (AgNPs) have attracted considerable attention in terms of their antimicrobial, medical, and chemical applications due to their high resistance to oxidation and high thermal conductivity.^25–32^ Furthermore, AgNPs can damage the bacterial cell membrane, disturb DNA replication, and lead to increased permeability and ultimately to cell death.^33–35^ The grain size of AgNPs is an important factor in this process.^36–38^

Recently, the antibacterial activity of graphene-based nanocomposites has attracted much attention due to the unique properties of graphenes; *e.g.*, their high theoretical specific surface.^39–41^ In this study, we report the fabrication of novel nanocomposites, namely GO decorated with Ag, TiO_2_, and ZnO nanoflowers (GO–Ag–TiO_2_@ZnO). For comparison, GO–Ag and GO–TiO_2_@ZnO have been fabricated and characterized. Several considerations led us to design the GO–Ag–TiO_2_@ZnO nanocomposite: (1) GO has the advantages of ease of fabrication, ease of processing, and economic production, with large scale and low cost; (2) GO has mild cytotoxicity to mammalian cells in low dose; (3) GO exhibits high antibacterial efficiency in its ability to damage the cell membranes *via* the generation of reactive oxygen species (ROS) and has exceptionally sharp edges; (4) Ag nanoparticles can consistently cause bacterial cell membrane damage, disturbing DNA replication, and leading to increased permeability and ultimately cell death;^42–46^ (5) TiO_2_ could be used for the killing or growth inhibition of bacteria due to its strong oxidation activity and super hydrophilicity; (6) ZnO exhibits antibacterial activity through generation of ROS and/or accumulation of NPs in the cytoplasm that lead to the interruption and inhibition of membrane and cellular tasks.

In order to confirm the antibacterial activity of the fabricated nanocomposites, the fluorescence quenching of bovine serum albumin (BSA) and circulating tumor DNA (ctDNA) were examined using the steady-state fluorescence technique. The results showed the excellent ability of the fabricated nanocomposite (GO–Ag–TiO_2_@ZnO) against growth of bacteria by destroying the DNA bacteria; this was confirmed with both Gram-positive and Gram-negative bacteria.

## 2. Experimental section

### 2.1. Chemicals and materials

Silver nitrate, zinc acetate, titanium(iv) *n*-isobutoxide, graphite, potassium hydroxide, and ethanol were purchased from Sigma-Aldrich. All used chemicals in this study were of reagent grade and were used without any further purification.

#### 2.1.1. Synthesis of graphene oxide nanostructure

A modified Hummers and Offeman's method was used to fabricate the GP nanosheet from natural graphite powder.^47,48^ 8 g of graphite powder (Sigma-Aldrich) and a stoichiometric amount of NH_4_NO_3_ were added to 368 ml of 98% (w/w) H_2_SO_4_ in an ice bath. KMnO_4_ was slowly added to the mixture with continuous stirring until the solution turned green. 640 ml of pure water was added, and stirring continued at 90 °C until the solution yielded a brown precipitate. Finally, H_2_O_2_ (30%) was added slowly until the solution turned a yellow color. The solid was removed by filtration, washed with 10% HCl aqueous solution to remove metal ions, and washed several times with water. The GO obtained was dried at 45 °C.

#### 2.1.2. Synthesis of silver nanoparticles

In a three-necked glass flask, 1 mM of silver nitrate was dissolved in 100 ml deionized water (DW) under constant stirring and heating to 60 °C, then 1 mM of trisodium citrate was added as a stabilizing agent under stirring. 10% hydrazine hydrochloride as a reducing agent was added slowly with stirring, until a pale-yellow suspension formed, then the crystalline structure was centrifuged and washed several times with ethanol and water, and dried at 50 °C before undergoing analysis.

#### 2.1.3. Synthesis of titanium dioxide

6 ml of titanium(iv) *n*-isobutoxide 98% was added dropwise to a mixture of ethanol–water (4 : 1) at 90 °C. After undergoing reflux for 2 h at 90 °C, a white precipitate was produced. The obtained precipitate was centrifuged at 6000 rpm, and then washed several times with DW and ethanol, then dried at 50 °C and calcined at 470 °C for 2 h.^49^

#### 2.1.4. Synthesis of GO–Ag nanocomposite

0.04 g of GO nanosheet was dispersed in 100 ml water for 30 min by ultrasonication, and then heated to 60 °C. A solution of the fabricated silver NPs described previously was added dropwise, and a deep brown precipitate was formed. The precipitate was collected by centrifugation, then washed with H_2_O and ethanol, and the product was dried under vacuum at 45 °C.

#### 2.1.5. Synthesis of GO–TiO_2_@ZnO nanocomposite

0.04 g of GO nanostructure was dispersed in 100 ml water for 30 min by ultrasonication, and 100 ml aqueous solution of 0.2 M Zn(CH_3_COOH)_2_·2H_2_O was added slowly with stirring for 15 min. This mixture was then added dropwise to 1 M of a hot solution of KOH in ethanol. The resultant mixture was stirred for 3 h at 100 °C, and 0.5 g of TiO_2_ NPs was added to the mixture with vigorous stirring for 3 h. The resulting mixture was ultrasonication for 2 h, then the product was centrifuged and washed with water and methanol several times. GO–ZnO–TiO_2_@Ag nanocomposite was obtained after drying at 45 °C.

#### 2.1.6. Synthesis of GO–Ag–TiO_2_@ZnO nanocomposite

0.04 g of GO nanostructure was dispersed in 100 ml water for 30 min by ultrasonication and 100 ml aqueous solution of 0.2 M Zn(CH_3_COOH)_2_·2H_2_O was added slowly with stirring for 15 min. This mixture was added dropwise to 1 M of a hot solution of KOH in ethanol. The resultant mixture was stirred for 3 h at 100 °C, then 0.5 g of both Ag NPs and TiO_2_ NPs were added to the mixture with vigorous stirring for 3 h. The resultant mixture underwent ultrasonication for 2 h, then the product was centrifuged and washed with water and methanol several times. GO–ZnO–TiO_2_@Ag nanocomposite was obtained after drying at 45 °C.

### 2.2. Characterization techniques

Ultraviolet-visible (UV-vis) absorption spectra were measured using a Shimadzu UV-2450 spectrophotometer. Fluorescence spectra were recorded using a Shimadzu RF-5301PC spectrofluorometer. Fourier-transform infrared (FT-IR) spectra were recorded with a JASCO spectrometer 4100, using the KBr pellet technique. X-ray diffraction (XRD) measurements were reported using a Shimadzu 6000–XRD, X-ray diffractometer using Cu-Kα radiation 
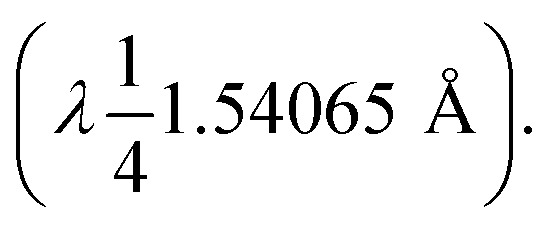
 Transmission electron microscopy (TEM) images were captured by a JEOL 2010 microscope operating at an accelerating voltage of 200 kV, and the morphology of the surface was estimated with scanning electron microscopy (SEM) using a JEOL (JSMIT100) instrument operating at 30 kV. Zeta potential results were carried out on Brookhaven zeta potential/particle size analyzer, and the surface area and pore size distribution were determined through Brunauer–Emmett–Teller (BET) analysis using Nova LX.^3^

### 2.3. Antimicrobial activity

#### 2.3.1. Preparation of microbiological cultures

For antibacterial assessment, *S. aureus* and *B. anthracoides* were selected as representative Gram-positive bacteria. *E. coli*, *P. multocida*, and *P. multocida* were selected as representative Gram-negative bacteria. All bacterial strains were obtained from the Central Diagnostic and Research Laboratory at the Faculty of Veterinary Medicine, Kafrelsheikh University, Egypt. At first, the tested bacterial strains were cultured on blood agar media (Oxoid) at 37 °C for 24 h. The strains of *S. aureus*, *B. anthracoides*, and *E. coli* were grown in Mueller–Hinton broth (Oxoid) media, while *P. multocida* was grown on tryptic soya broth (Oxoid). Briefly, 10 ml of the appropriate broth was inoculated with a single colony of each bacteria strain and incubated at 37 °C for 12 h.^50^

#### 2.3.2. Zones of inhibition

The disk diffusion (Kirby–Bauer) method was used to evaluate the antimicrobial activity of each nanoparticle compound. Bacterial cultures were diluted in the Mueller–Hinton broth, and achieved an optical density corresponding to 0.5 MacFarland standards, in which the concentration of bacteria was 1.5 × 10^8^ CFU ml^−1^. The Mueller–Hinton agar medium (Oxoid) was prepared and sterilized at 121 °C in an autoclave, then about 15 ml of the melted agar media was poured aseptically into each of the sterilized Petri plates and kept at room temperature for solidification. 100 μl of the prepared bacterial cell suspension was pipetted and spread across the dried surface of the Muller Hinton agar. Sterile filter paper disks loaded with each nanoparticle (at a concentration of 0.005 g/10 ml) were placed on the surface of the Mueller–Hilton agar plate using sterile forceps. The plates were incubated at 37 °C for 24 h. Following incubation, the zones of inhibition were measured in mm from four sides of each well to determine an average mean value. The presence of inhibition zones was measured by vernier caliper, and was recorded and considered as an indication for antibacterial activity.^51,52^

## 3. Results and discussion

### 3.1. XRD analysis

**Fig. 1:**
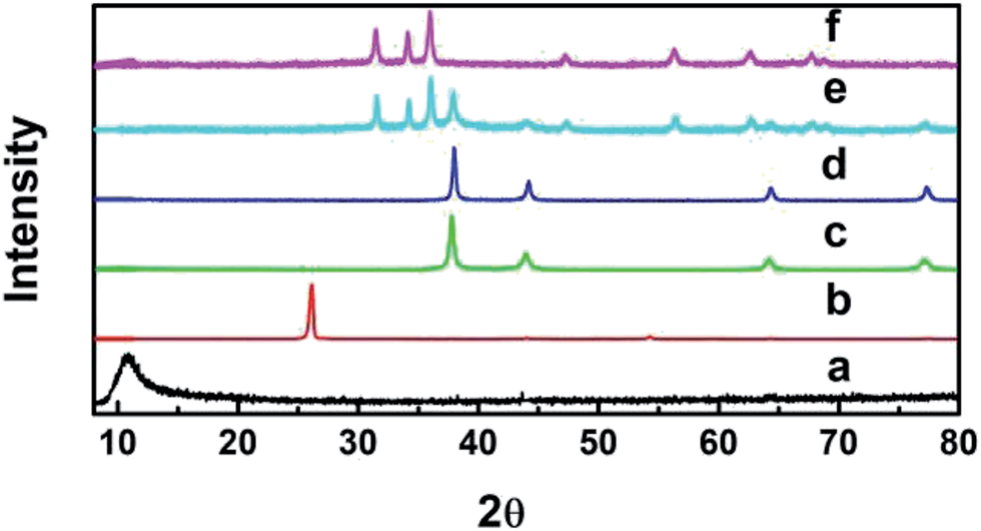
XRD patterns of (a) GO, (b) graphite, (c) Ag, (d) GO–Ag, (e) GO–Ag–TiO_2_@ZnO, and (f) GO–TiO_2_@ZnO.

Fig. 1 shows the patterns of the pure graphite powder, GO, Ag, GO–Ag, GO–TiO_2_@ZnO and GO–Ag–TiO_2_@ZnO nanocomposites. The diffraction peaks of pure graphite powder and GO were recorded at 26° and 10.9°, respectively, indicating the presence of oxygenated functional groups on carbon sheets of GO.^53,54^ Ag NPs exhibited four peaks at 2*θ* = 38.18°, 44.25°, 64.72°, and 77.4° that were attributed to the (111), (200), (220), and (311) crystalline planes.^55,56^ The presence of Ag nanoparticles over the GO surface in the nanocomposite (GO–Ag) was confirmed by recording peaks at 2*θ* = 37.7°, 44.3°, 64.0°, and 77.0°.^57,58^ GO–TiO_2_@ZnO nanocomposite showed the characteristic peaks of ZnP nanoparticles at 31.67°, 34.31°, 36.1°, 56.5°, 62.7°, and 67.9°,^59^ while the TiO_2_ pattern showed values for the diffraction peak at 25° and 47.59°.^60^ The slight shift to the lower region side might be due to the loading of TiO_2_ and ZnO NPs on the GO surface. GO–Ag–TiO_2_@ZnO nanocomposite showed a sharp highly intensive peak at 37.8°, 44°, 64.2°, and 77.2° (for Ag), 47.3° (for TiO_2_), and 31.67°, 34.31°, 36.1°, 56.39°, 62.7°, and 67.9° (for ZnO). These features suggest that the metal oxides (ZnO, TiO_2_) and Ag NPs are loaded on the GO surface. The absence of a typical peak for GO may be due to the disruption and good exfoliation of GO in the nanocomposites and/or the loading of metal oxide NPs into the surface of GO oxide.

The main diameter was calculated from Debye–Scherrer's® formula (eqn (1)):

1 *D* = *Kλ*/*β* cos *θ*

where *K* is a constant representing shape factor (∼0.9), *λ* is the wavelength of the X-ray source (1.5405 Å), *β* is the full width at half maximum of the diffraction peak, and *θ* is the angular position of the peak. According to this equation, the average diameters of Ag NPs and GO–Ag were determined to be ∼20–30 nm and 58 nm, respectively. In GO–TiO_2_@ZnO, the average diameters were found to be ∼48 nm (for ZnO) and ∼35 nm (for TiO_2_). When turning to GO–Ag–TiO_2_@ZnO, the average diameters were determined to be ∼24–40 nm (for Ag), ∼40–58 nm (for ZnO) and ∼24–40 nm (for TiO_2_). The results illustrate that the anchoring of GO with metal oxide NPs has little influence on the crystallite size of the phase structure of the metal oxide NPs.

### 3.2. Scanning electron microscopy analysis

**Fig. 2:**
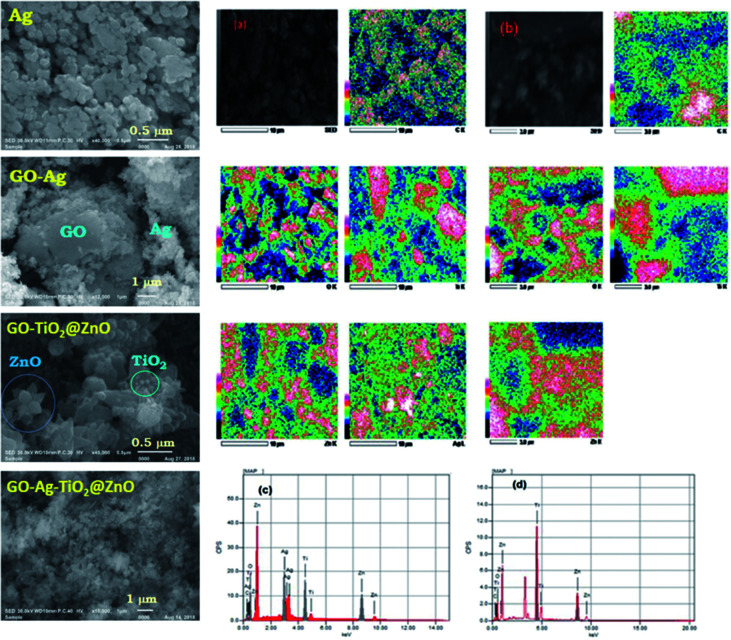
(Left) SEM images of Ag, GO–Ag, GO–TiO_2_@ZnO, and GO–Ag–TiO_2_@ZnO. (Right) SEM mapping of (a) GO–Ag–TiO_2_@ZnO and (b) GO–TiO_2_@ZnO. (Bottom right) EDX of mapping image of (c) GO–Ag–TiO_2_@ZnO and (d) GO–TiO_2_@ZnO.

SEM was performed with different magnifications to exhibit the morphology and energy-dispersive X-ray (EDX) of the metal oxides. Fig. 2 shows SEM images of GO, Ag, GO–Ag, GO–TiO_2_@ZnO, and GO–Ag–TiO_2_@ZnO nanocomposites. As can be seen, the surface of the GO nanosheet was densely packed by metal oxides, indicating a good combination between GO and the metal oxide NPs. The GO nanosheets seem to act as bridges for the metal oxide entities. Ag NPs have a spherical morphology and are loaded successfully on the GO surface. As shown in Fig. 2c, the ZnO nanoflower appears clearly over the GO surface, while TiO_2_, with its spherical shape, is deposited on the ZnO nanoflower. This matching between the deposited ZnO and TiO_2_ over the GO surface confirmed by EDX formation of the GO–TiO_2_@ZnO nanocomposite. The GO–Ag–TiO_2_@ZnO nanocomposite presented as shown in Fig. 2, right. The GO–TiO_2_@ZnO and GO–Ag–TiO_2_@ZnO nanocomposites were analyzed using EDX with uniform particle morphology (Fig. 2, right).

### 3.3. UV-vis absorption studies

The absorption spectra of the examined nanocomposites and the control compounds were recorded in water at room temperature, as shown in Fig. S1.† The absorption spectrum of GO exhibited a strong absorption peak at 228 nm and shoulder at 300 nm, which was assigned to the π–π* transitions of the aromatic C=C bonds and n–π* transitions of C=O bonds, respectively.^61,62^ Ag NPs and GO–Ag exhibited absorption bands at 440 and 420 nm that were assigned to the surface plasmon resonance of Ag NPs. This arises from the interaction of the incident light with the valence electrons of Ag NPs, leading to the oscillation of electrons along with the frequency of the electromagnetic source.^63^ GO–TiO_2_@ZnO exhibited absorption peaks at 365 nm (ZnO), 244 nm (TiO_2_), and 290 nm (GO). For GO–Ag–TiO_2_@ZnO, absorption peaks were visible at 372 nm (ZnO), 437 nm (Ag), 250 nm (TiO_2_), and 290 nm (GO). The energy band gap values of the nanocomposites were determined by the Tauc equation (eqn (2)):^64,65^

2 *αhν* = *A*(*hν* − *E*_g_)^*n*^

where *α* is the absorption coefficient, *h* is Planck's constant, *ν* is the frequency of light, *A* is a proportionality constant, *E*_g_ is the band gap and *n* = 1/2 for the direct transitions.^66^ A plot of (*αhν*)^2^*versus hν* is shown in the inset of Fig. S2 in the ESI.† The linear portion of the curve is extrapolated to the *hν* axis to determine the energy gap. The band gap values were found to be 4.00, 2.99, 2.80, 2.62, and 2.50 eV for GO, Ag, GO–Ag, GO–TiO_2_@ZnO, and GO–Ag–TiO_2_@ZnO, respectively.

### 3.4. Zeta potential analysis

**Fig. 3:**
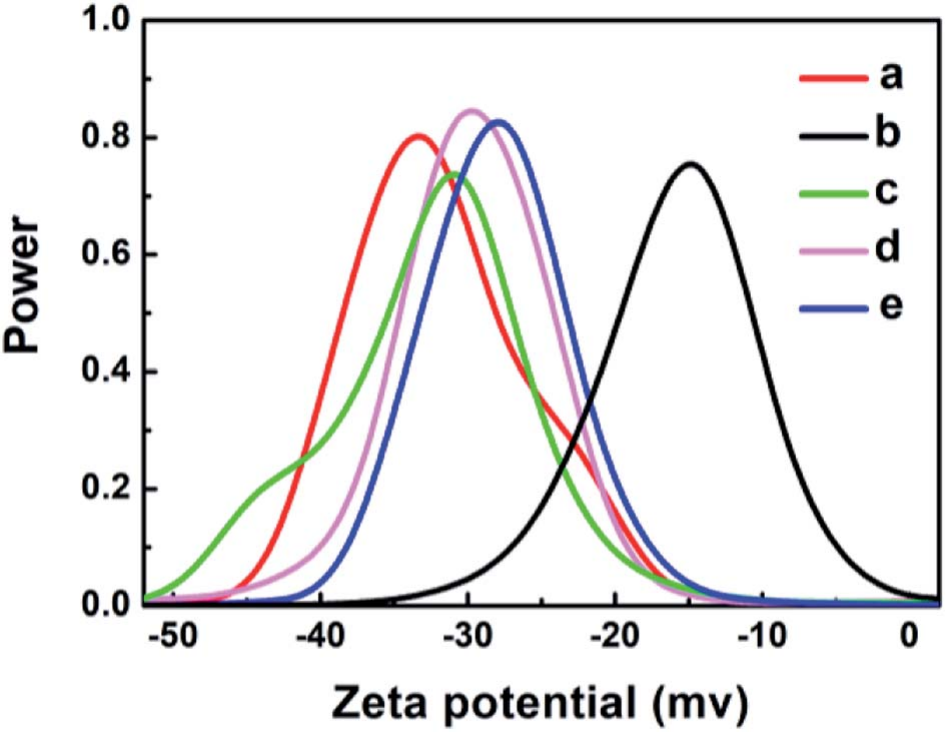
Zeta potential graphs of: (a) GO, (b) Ag, (c) GO–Ag, (d) GO–TiO_2_@ZnO, and (e) GO–Ag–TiO_2_@ZnO in water.

The zeta potential technique was used to assess the stability of the nanoparticles and nanocomposites in solution and to understand the charge on the surface. Fig. 3 shows that the particles are negatively charged for GO (−33 mV), Ag (−15 mV), GO–Ag (−31 mV), GO–TiO_2_@ZnO (−29 mV), and GO–Ag–TiO_2_@ZnO (−27 mV). The negative values suggest the higher stability of colloidal dispersions of particles in water. Moreover, the zeta potential was measured at room temperature in DW as a medium. Compared with Ag NPs, the nanocomposites were highly dispersed in water, indicating the effect of GO in increasing the stability of metal oxide NPs in solution.

### 3.5. FT-IR analysis

**Fig. 4:**
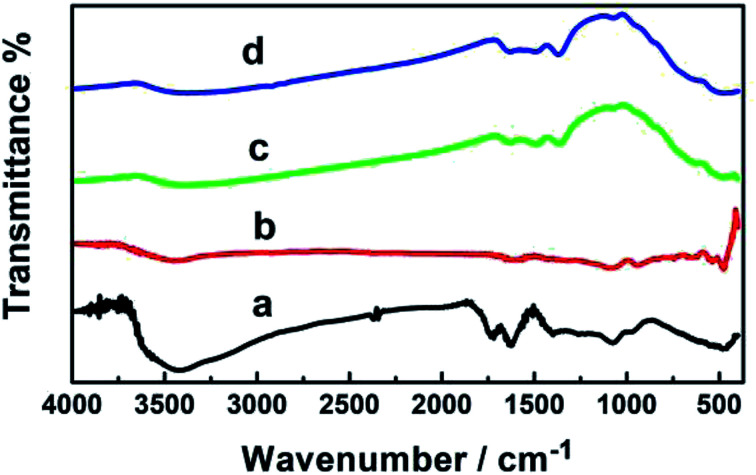
FT-IR spectra of (a) GO, (b) GO–Ag, (c) GO–Ag–TiO_2_@ZnO, and (d) GO–TiO_2_@ZnO.

Fig. 4 shows the FT-IR spectra of the fabricated GO, GO–Ag, GO–TiO_2_@ZnO, and GO–Ag–TiO_2_@ZnO. GO exhibited two bands at 3400 and 1620 cm^−1^ that were assigned to the stretching vibration of the (OH) group and the skeletal vibration of the graphene sheets, respectively. Strong peaks at 1730, 1370, 1220, 1165, and 1058 cm^−1^ were assigned to the stretching vibration of oxygen-containing carboxyl (C=O), carboxyl (C–O), epoxy (C–O), carboxyl (C–OH), and alkoxy (C–O) functional groups, respectively.^67–69^ This indicates numerous oxygen-containing functional groups over the GO surface. The characteristic peaks of GO–Ag in the adsorption band at approximately 1629 cm^−1^ correspond to the C=C bonding of the aromatic rings of the GO carbon skeleton structure. The presence of other oxygenated functional groups was observed for OH (at 3437 and 1427 cm^−1^), C=O (at 1629 cm^−1^), C–OH (at 1335 cm^−1^), and C–O (at 1084 cm^−1^).

In the GO–Ag nanocomposite, the significant decrease in the absorption bands of the oxygenated functionality could be explained by the existence of AgNPs over the surface of GO.^70,71^ GO–TiO_2_@ZnO usually showed characteristic absorption bands at 523 and 638 cm^−1^ due to two transverse optical stretching modes of ZnO and TiO_2_, respectively. The recorded band at 3435 cm^−1^ can be assigned to the stretching vibration of the surface hydroxyl (–OH) groups on the surface of the metal oxide nanoparticles. These changes in functional group suggest that ZnO and TiO_2_ NPs were successfully immobilized on the surface of GO by recording the bands at 456 and 566 cm^−1^.^72,73^ GO–Ag–TiO_2_@ZnO nanocomposite showed characteristic peaks of ZnO (at 484 cm^−1^), TiO_2_ NPs (at 584 cm^−1^), and the stretching vibration of the surface hydroxyl (–OH) groups on the surface metal oxide nanoparticles (at 3435 cm^−^^1^).

### 3.6. Brunauer–Emmett–Teller theory (BET) analysis

**Fig. 5:**
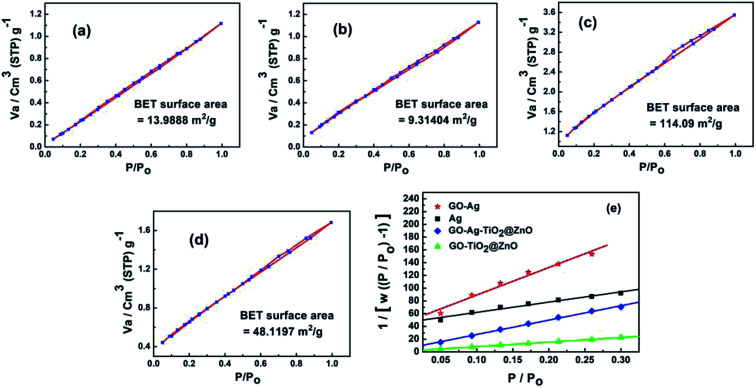
N_2_ adsorption/desorption isotherm curve of (a) Ag, (b) GO–Ag, (c) GO–TiO_2_@ZnO, and (d) GO–Ag–TiO_2_@ZnO nanocomposites. (e) Langmuir fits from the N_2_ adsorption data for Ag, GO–Ag, GO–TiO_2_@ZnO, and GO–Ag–TiO_2_@ZnO nanocomposites.

Fig. 5 shows the surface area and pore size of the fabricated nanoparticles using the nitrogen adsorption–desorption full isotherm. The loop of isotherm was of type (IV) with a H1 hysteresis loop (0.4 < *P*/*P*_0_ > 0.95) indicating that the surfaces of these materials have a high degree of pore size uniformity. The results illustrated that the surface has one type of pore, which is mesoporous with a diameter of 2–50 nm. The surface area values for GO, Ag NPs, GO–Ag, GO–TiO_2_@ZnO and GO–Ag–TiO_2_@ZnO nanocomposites were determined to be 253.87, 13.98, 9.31, 114.10, and 48.12 m^2^ g^−1^, respectively. The average pore size was determined to be 1.223, 2.238, 2.315, 1.898, and 2.047 nm for GO, Ag, GO–Ag, GO–TiO_2_@ZnO, and GO–Ag–TiO_2_@ZnO nanocomposites, respectively (Fig. S2†).

### 3.7. Antimicrobial activity

**Fig. 6:**
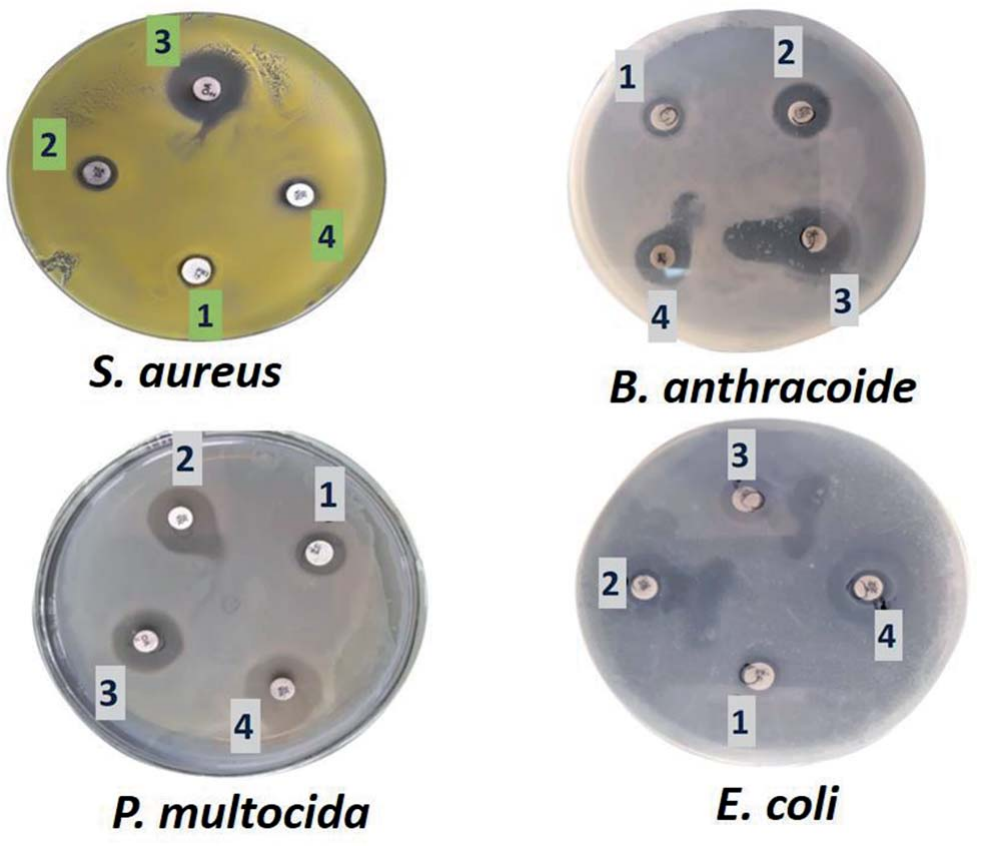
Effect of Ag (1), GO–Ag (2), GO–TiO_2_@ZnO (3), and GO–Ag–TiO_2_@ZnO (4) on Gram-positive and Gram-negative bacteria.

**Scheme 1:**
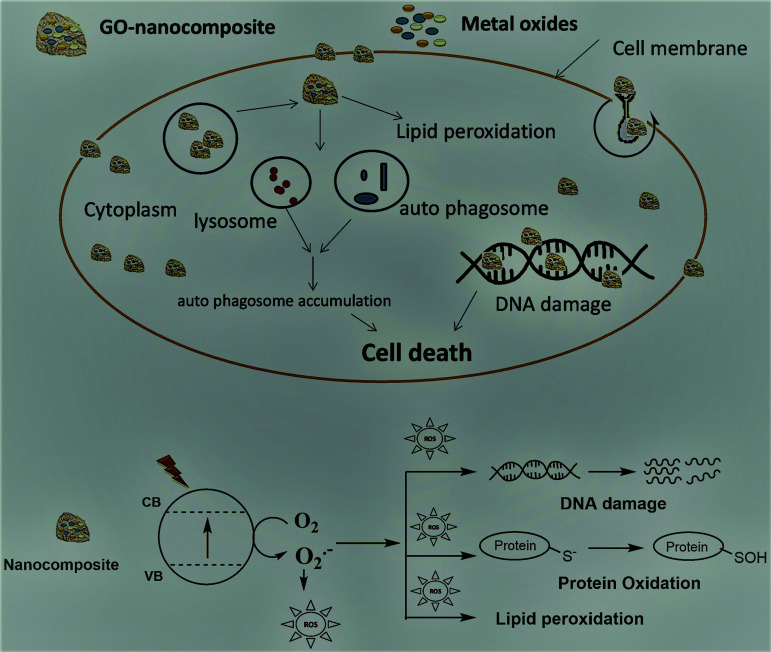
Mechanism of antibacterial activity.

The fabricated GO–Ag, GO–TiO_2_@ZnO, and GO–Ag–TiO_2_@ZnO (0.005 g/10 ml water) nanocomposites were used to investigate their antibacterial activity against different types of bacteria that included strains of Gram-positive bacteria (*S. aureus* and *B. anthracoides*) and strains of Gram-negative bacteria (*E. coli* and *P. multocida*) by using the disc-diffusion method. The results reveal that all nanocomposites were potentially effective in suppressing bacterial growth with variable potency. GO–TiO_2_@ZnO nanocomposite showed highly suppressing microbial growth against both Gram-positive and Gram-negative bacteria, but a more marked effect against Gram-negative bacteria was observed compared with Gram-positive bacteria. The Ag NPs alone had the lowest inhibitory effect on all tested bacteria. With regards to the effect of GO–Ag–TiO_2_@ZnO and GO–Ag on tested bacteria, the obtained results showed marked suppression of this nanocomposite against Gram-negative bacteria, However, its effect on tested Gram-positive bacteria varied, in that *B. anthracoides* was more sensitive to GO–Ag–TiO_2_@ZnO than *S. aureus* (Fig. 6). The overall results revealed that these nanocomposites are more effective against Gram-negative bacteria than Gram-positive bacteria. The differences in the effects of various nanoparticles on examined bacteria might be correlated with a number of factors, including type of bacteria examined, type of manufactured nanoparticle, and methodology used. The mechanism of the antibacterial effect by the fabricated nanocomposites is summarized in Scheme 1. It is most likely that the higher antibacterial activity of the GO–Ag–TiO_2_@ZnO nanocomposite arises from the combination between the direct damage of the cellular membranes by Ag NPs, the generation of ROS by the TiO_2_ and ZnO entities, and the accumulation of NPs in the cytoplasm.^75–78^

### 3.8. Fluorescence quenching spectra

**Fig. 7:**
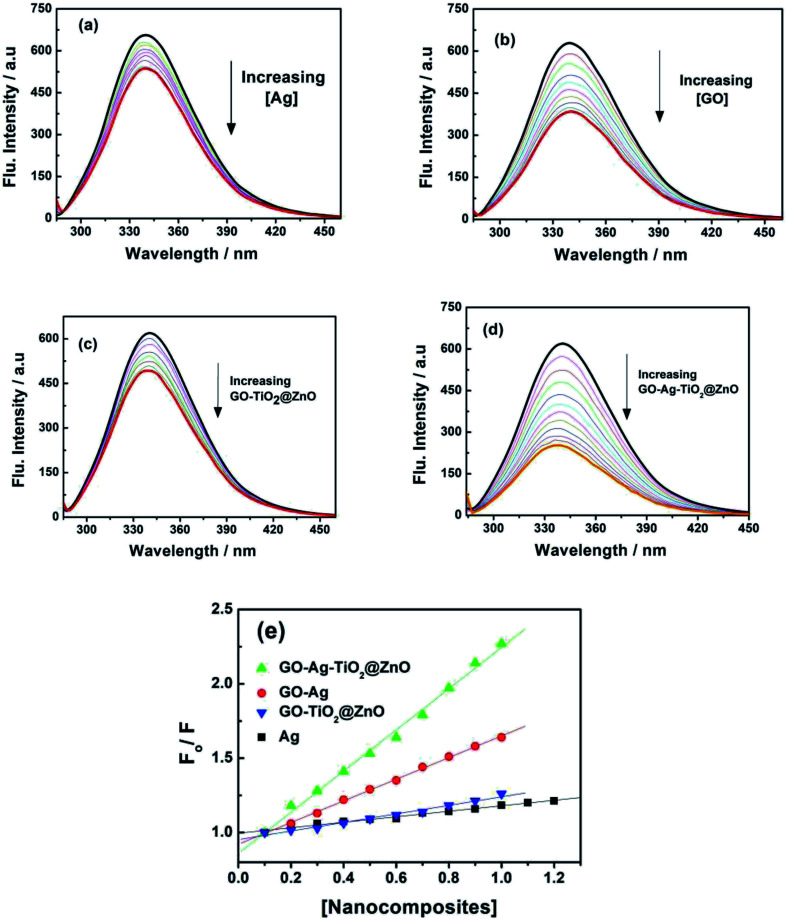
Fluorescence spectra of BSA with different concentration of (a) Ag, (b) GO–Ag, (c) GO–TiO_2_@ZnO, and (d) GO–Ag–TiO_2_@ZnO at *λ*_ex_ = 280 nm. (e) Stern–Volmer plots.

**Fig. 8:**
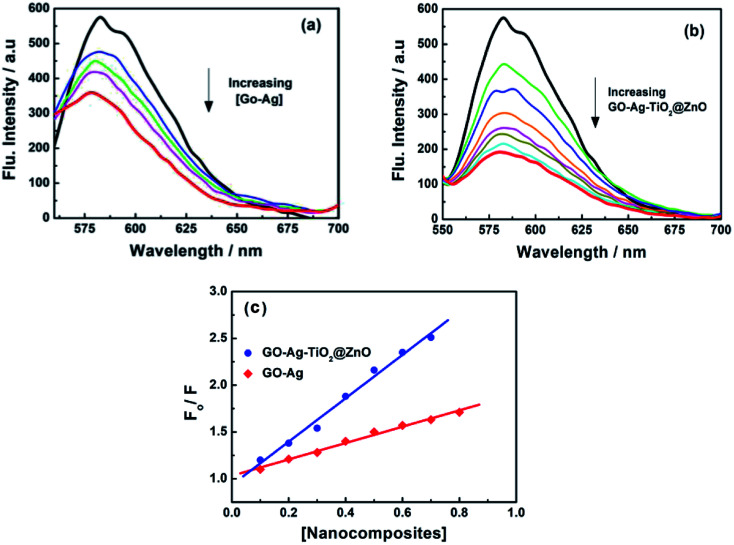
Fluorescence quenching of ctDNA with different concentrations of (a) GO–Ag and (b) GO–Ag–TiO_2_@ZnO in Tris–HCl buffer; *λ*_ex_ = 260 nm; (c) Stern–Volmer plots.

To support the suggested mechanism shown in Scheme 1, the interaction between the fabricated nanocomposites and the protein and DNA was examined using fluorescence measurements. Fig. 7a–d shows the steady-state fluorescence quenching process of BSA with different concentrations of Ag, GO–Ag, GO–Ag–TiO_2_@ZnO, and GO–TiO_2_@ZnO nanocomposites in water. Upon excitation with 280 nm, the emission of a BSA singlet state at 340 nm was considerably quenched in the presence of the examined nanocomposites. Stern–Volmer plots showed a higher quenching rate constant in the case of GO–Ag–TiO_2_@ZnO compared with control nanocomposites (Ag, GO–Ag, and GO–TiO_2_@ZnO) (Fig. 7e).^74^ The quenching constants of the singlet BSA in the presence of different amounts of Ag, GO–Ag, GO–TiO_2_@ZnO, and GO–Ag–TiO_2_@ZnO nanocomposites were found to be 0.18, 0.73, 0.28, and 1.38 min^−1^, respectively. Similar observations were recorded by following the fluorescence quenching of ctDNA by adding different amounts of the examined nanocomposites in Tris–HCl buffer (Fig. 8). The quenching rate constants of the singlet ctDNA with the addition of GO–Ag and GO–Ag–TiO_2_@ZnO nanocomposites were found to be 0.874 and 2.32 min^−1^, respectively.

## 4. Conclusion

We report herein the fabrication of promising nanocomposites, namely GO–Ag, GO–TiO_2_@ZnO, and GO–Ag–TiO_2_@ZnO. The fabricated nanocomposites were characterized using different analytical and spectroscopic techniques (XRD, SEM, and FT-IR). Steady-state fluorescence measurements provided clear evidence of the fluorescence quenching of BSA and ctDNA in the presence of different amounts of the fabricated nanocomposites. An antibacterial test was performed with two Gram-positive and two Gram-negative bacteria. GO–TiO_2_@ZnO nanocomposite was the most effective in suppressing microbial growth of the tested Gram-positive bacteria, while it had a moderate effect against Gram-negative bacteria. GO–Ag and GO–TiO_2_@ZnO nanocomposites showed strong activity against both Gram-positive and Gram-negative bacteria, and these nanocomposites are more effective against Gram-negative bacteria than Gram-positive bacteria. GO–Ag, GO–TiO_2_@ZnO, and GO–Ag–TiO_2_@ZnO nanocomposites have antibacterial activity against Gram-negative and Gram-positive bacteria, with Ag NPs alone having the lowest inhibitory effect on all tested bacteria.

## Conflicts of interest

The authors have no conflicts to declare.

## Supplementary Material

RA-009-C8RA09788G-s001
