# Supplementary material for: Graphene oxide decorated with zinc oxide nanoflower, silver and titanium dioxide nanoparticles: fabrication, characterization, DNA interaction, and antibacterial activity
Source: RSC Adv. 2019 Jan 28;9(7):3704–14. doi: 10.1039/c8ra09788g (PMC9060286; doi:10.1039/c8ra09788g)
Supplement: RA-009-C8RA09788G-s001 [file RA-009-C8RA09788G-s001.pdf]

Electronic Supplementary Material (ESI) for RSC Advances.
This journal is © The Royal Society of Chemistry 2019

For RSC Advances

Graphene oxide nanosheet decorated with zinc oxide nanoflower, silver and titanium dioxide NPs: Fabrication, characterization, DNA interaction, and antibacterial activity

Nagi El-Shafai,[ab] Mohamed El-Khouly,*[c,d] Maged El-Kemary,[bc] Mohamed Ramadan,[a] Ibrahim Eldesoukey,[e] and Mamdouh Masoud[a]

[a] Department of Chemistry, Faculty of Science, Alexandria University, Egypt

[b] Institute of Nanoscience and Nanotechnology, Kafrelsheikh University, Kafr El-Sheikh 33516, Egypt

[c] Chemistry department, Faculty of Science, Kafrelsheikh University, Egypt

[d] Institute of Basic and Applied Sciences, Egypt-Japan Institute of Science and Technology, Alexandria, Egypt

[e] Bacteriology, Mycology and Immunology department, Faculty of Veterinary Medicine, Kafr El-Sheikh University 33516, Egypt

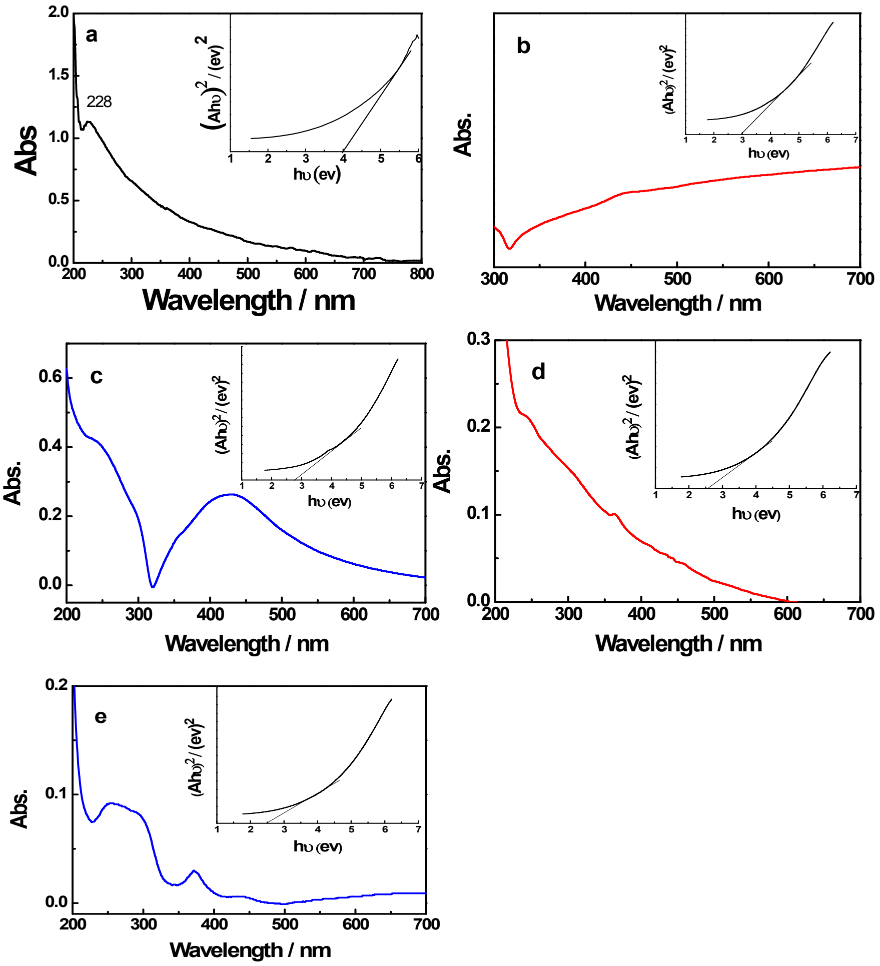

Fig. S1 UV-vis spectra and energy gap of GO (a) GO-CuO (b), CuO (c), GO-CuO@ZnO (d) and ZnO (e).

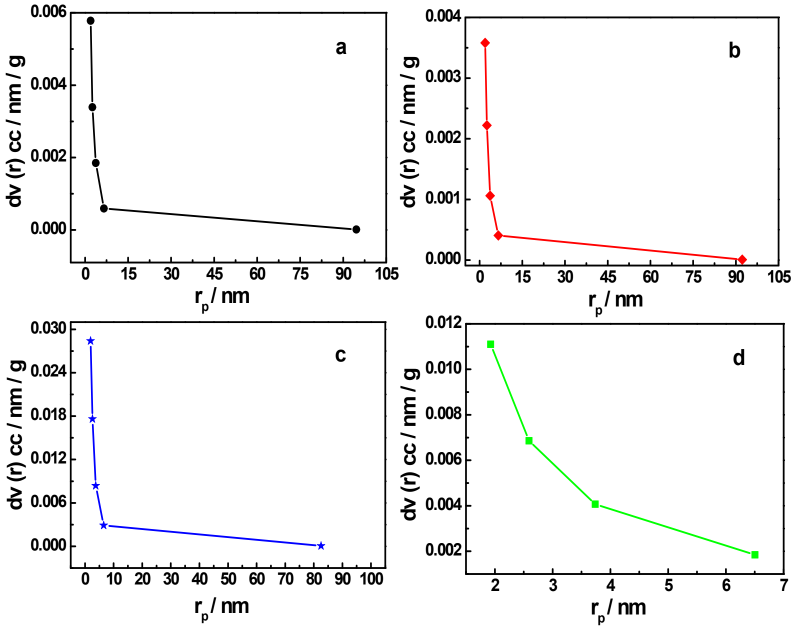

Fig. S2. BJH Pore size distribution of Ag (a) GO-Ag (b) GO-TiO_2@ZnO (c), and GO-Ag-TiO_2@ZnO (d) nanocomposites.
